# Changes of Altruistic Behavior and Kynurenine Pathway in Late-Life Depression

**DOI:** 10.3389/fpsyt.2020.00338

**Published:** 2020-04-30

**Authors:** Yujie Wu, Naikeng Mai, Xuchu Weng, Jiuxing Liang, Yuping Ning

**Affiliations:** ^1^School of Psychology, South China Normal University, Guangzhou, China; ^2^Department of Neurology, The Affiliated Brain Hospital of Guangzhou Medical University (Guangzhou Huiai Hospital), Guangzhou, China; ^3^Institute for Brain Research and Rehabilitation, South China Normal University, Guangzhou, China; ^4^The First School of Clinical Medicine, Southern Medical University, Guangzhou, China; ^5^Guangdong Engineering Technology Research Center for Translational Medicine of Mental Disorders, Guangzhou, China

**Keywords:** altruism, kynurenine pathway, late-life depression, Dictator Game, diffusion tensor imaging, graph-theoretical analysis

## Abstract

**Background:**

Depressive patients show less altruistic behavior. While, older adults present higher tendencies for altruism than younger adults. Depression and age are two of the influencing factors of altruism, kynurenine (KYN), and its metabolites. However, the characteristics of altruism in late-life depression (LLD) and its possible underlying mechanism have not been studied.

**Objective:**

We aimed to explore the characteristics of altruism in LLD patients and its neurobiological mechanism and structural brain network. We investigated whether the levels of metabolites in kynurenine pathway (KP) and white matter (WM) network topological features would influence the altruistic behavior in LLD patients.

**Methods:**

Thirty-four LLD patients and 36 heathy controls (HCs) were included. Altruism was evaluated by the Dictator Game (DG) paradigm. Serum concentrations of KP metabolites were detected by the liquid chromatography-tandem mass spectrometry method. The topological features of the WM network were calculated from diffusion tensor imaging data in conjunction with graph-theoretical analysis.

**Results:**

The LLD participants exhibited a higher level of altruism and WM global network properties than the HCs. Kynurenic acid to kynurenine (KYNA/KYN) ratio was associated with the DG performance in LLD group. KYNA/KYN ratio was associated with the WM network properties in HC group.

**Conclusions:**

KYN metabolism played an important role in altruistic behavior in LLD.

## Introduction

Depression is the most common mental disorder in the growing geriatric population (1). Late-life depression (LLD) is associated with physical and cognitive deficits and the poor treatment responses of these patients, which results in a heavy economic and emotion burden to their families (2–5). Moreover, deficits of social functioning commonly occur in patients with depression, which have an impact on their work performances and marriages (6). However, seldom investigations shed light on social impairments in LLD. Hence, we attempted to focus on the social functioning in LLD from the perspective of neural, biological, and behavioral factors.

Altruistic behavior is a kind of typical prosocial behavior in human societies, influencing the interactions and cooperations among individuals (7). Altruism is a characteristic in which people are willing to help others even when there is no expectation of receiving any help or reward in return. Altruism has been modeled using the Dictator Game (DG) (8). Based on previous studies, depression and age are two of the primary influencing factors of altruistic behavior. Depression patients at midlife would avoid altruistic behaviors (9). Furthermore, older adults show higher tendencies for altruism than younger adults (10). However, altruistic behavior in depression in old age and its possible underlying mechanism have not been studied.

There are many possible factors can influence altruistic behavior, such as social environment, economic condition and hormones (11). According to a literature review, 5-hydroxytryptamine (5-HT) plays an important role in altruism. Depletion of 5-HT is associated with decreased activity in the striatum by affecting dopaminergic terminal function (6). While, the level of dopamine in the ventral striatum is one of vital factors that influences altruism decision (8). Kynurenine pathway (KP) plays an important role in 5-HT synthesis. Kynurenine (KYN) and its metabolites can cross the blood-brain barrier and have effects on the central nervous system and several psychiatric disorders, such as depression and schizophrenia (12). Tryptophan (TRP) is considered as the beginning of KP. The shunt of TRP from 5-HT formation to KYN formation is a major etiological factor of depression (13, 14). We aimed to explore the relationship between several main metabolites in the upstream and downstream of KP and altruistic behavior in patients with LLD.

KP changes are associated with the development of LLD (15). There are two distinct routes in KYN metabolism. One such route is the kynurenic acid (KYNA) pathway. The other is the quinolinic acid (QUIN) pathway, which forms the N-methyl-D-aspartate receptor (NMDA-R) agonists that make the astrocyte-microglia-neuronal network vulnerable. KYNA exerts a neuroprotective role and is the only known endogenous antagonist to NMDA-R, which can inhibit the toxic effect of QUIN (16). However, the abnormal accumulation of KYNA beyond physiological levels could induce glutamatergic hypofunctioning and cause cognitive dysfunction, since KYNA is an antagonist for all of the ionotropic excitatory amino acid receptors (17). The severity of depression is associated with the level of KP imbalance (18). TRP metabolism has been related to depressive symptoms in old age (19). According to our consideration, if LLD patients display different altruism levels, KP alterations, especially the KYN metabolism changes, may represent one neurobiological explanation and associated with the level of altruism.

Previous studies mention that KP is associated with the disrupted white matter (WM) in bipolar disorders (20), schizophrenia (21), and multiple sclerosis (22). However, the study on the relationship between KP and changes of WM in LLD is not been found. Some scholars have found that LLD is characterized by WM lesions that affect the WM tract integrity and alter the rich-club organization, which disrupt cognition function and mental health in LLD patients (23, 24). In addition, we suspected that KP possibly has an impact on the WM connectivity in LLD, which may underlie the development of the cognitive and social functioning deficits of LLD. Furthermore, the neural pathway of altruistic behavior is not fully understood. According to the previous study of functional connectivity, the engagement of the medial prefrontal and temporo-parietal cortices is associated with prosocial behavior (25). While the changes of function is related with the changes of structure (26). Thus, we considered the changes of altruistic behavior in LLD might blame to some abnormal brain structural network connectivity.

Diffusion tensor imaging (DTI), a technique that is used to quantify water diffusion in tissue, is sensitive to tissue damage and can detect damage in the WM (23). The human brain serves as a highly complex and integrated network system. The graph-theoretical analysis of this complex network has resulted in a potent mathematical tool to quantify the collection of the comprehensive topological dynamics in these human structural connectomes (27). Considering the light of corresponding relationship between the function and structure of our brain (26), the structural foundation of the altruism changes that occur in older people is another question we want to investigate. DTI data in conjunction with graph-theoretical analysis makes it possible to quantitatively describe the brain's overall organization and communicative processes through a variety of physical topological properties.

In this study, we aimed to investigate the characteristics of altruistic behavior, KP metabolism, and WM network connectivity in LLD, and further explore whether KP metabolism and WM network topological features would influence altruistic behavior in LLD patients. We collected DG performance, serum sample, and DTI data from each participant. The DG task was conducted to assess altruistic behavior. Serum concentrations of TRP, KYN, and KYNA were determined by a high-performance liquid chromatography-tandem mass spectrometry (LC-MS/MS) method. A graph-theoretical analysis was utilized to calculate the global network properties that describe the WM topological features. We hypothesized that disruptions of KP metabolism and WM network topological features would occur in patients with LLD, and these disruptions would relate to altruism in LLD.

## Materials and Methods

### Participants

This study was approved by the ethics committee of the Affiliated Brain Hospital of Guangzhou Medical University (Guangzhou Huiai Hospital). We obtained written informed consent from each participant after a complete description of this study. All of the patients were recruited from the outpatient and inpatient departments of the Affiliated Brain Hospital of Guangzhou Medical University, Guangzhou, Guangdong, China, and the healthy controls (HCs) were recruited from the community.

All participants were interviewed by well-trained psychiatrists during a clinical interview that was structured to meet the inclusion and exclusion criteria. LLD was diagnosed based on *Diagnostic and Statistical Manual of Mental Disorders, fourth edition*. Cognition performance was evaluated by a Mini-Mental State Examination (MMSE). The presence of depressive symptoms was evaluated by the 17-item Hamilton Rating Scale for Depression (HAMD-17). Exclusion criteria were as follows: (1) serious suicidal behavior; (2) serious medical conditions or concomitant medications likely to influence the central nervous system or immunological function, including cardiovascular, respiratory, endocrine and neurological diseases; (3) a history of drug or alcohol abuse in the past 6 months or a history of drug or alcohol dependence within the past year; (4) < 55 years old. In addition, the gender- and age-matched HCs were required to have no first-degree relative with a psychiatric disorder (15, 28).

Finally, we recruited investigated 34 patients with a diagnosis of LLD and 36 healthy elderly subjects as HCs, and all of the participants were of Han Chinese ethnicity and were right-handed.

### Experimental Procedures

Upon participants' arrival to the outpatient department, we collected peripheral blood samples and measured the body mass index (BMI) of every participant between 8:00 and 9:00 a.m. after an overnight fast. The blood samples were collected in vacutainers without additional additives. After 0.5 h of coagulation, the samples were centrifuged at 3,000 r/min for 10 min, and the supernatant was aliquoted into Eppendorf tubes (Eppendorf, Hamburg, Germany) and frozen at −80°C immediately until the time of the assay. Then, the DG task and neuropsychological tests were performed.

In DG procedures, we told participant that there were other participants who also took part in this game simultaneously but they did not know each another. The another old person played a passive recipient in DG task, and the participant played a proposer could choose how to allocate 1,000 monetary units (Yuan) between himself/herself and another old person. In the current study, DG task was anonymous paradigm in case of bias choice. Each participant played 30 rounds one-shot DG task against 30 anonymous players. With no material incentive to offer anything, a proposer who offers a nonzero amount is considered to be altruistic and the magnitude of their proposal reflects the degree of altruism from the proposer toward the passive recipient (8).

### Laboratory Analyses

We used an LC-MS/MS method to detect the TRP, KYN, and KYNA serum concentrations. L-TRP, L-KYN, KYNA, and activated charcoal were purchased from Sigma-Aldrich, and Kyna-d5 was supplied by Toronto Research Chemicals, Inc. (Toronto, Canada). Methanol was obtained from Merck KGaA (Darmstadt, Germany) and ammonium formate was purchased from Sigma-Aldrich Corporation (Bangalore, India). Purified water was produced by a Milli-Q water purification system (Millipore Corporation, Billerica, MA, USA) (29).

### MRI Acquisition

Participants were scanned by a 3.0-Tesla Philips Achieva scanner (Philips, Best, Netherlands). The T2-weighted image was applied to rule out cerebral infarction, tumors, and major WM lesions. Foam padding and earplugs were used to reduce head moving and scanner noise. The DTI scanning parameters were as follows: direction = 32, b_0_ = 1,000 s/mm^2^, repetition time (TR) = 10,015 ms, echo time (TE) = 92 ms, flip angle = 90°, matrix = 128 × 128 mm^2^, FOV = 256 × 256 mm^2^, 75 contiguous slices, voxel size = 2 × 2 × 2 mm^3^. High-resolution T1-weighted images were acquired from a 3D spoiled gradient echo sequence: TR = 8.2 ms, TE = 3.8 ms, matrix= 256 × 256 × 188, FOV = 256 × 256 mm^2^, voxel size = 1 × 1 × 1 mm^3^.

### Data Preprocessing

Data preprocessing was performed using the FMRIB's Diffusion Toolbox (FMRIB's Software Library, FSL) for the following procedures. First, the eddy current correction was used to correct the distortions from stretches and shears in the DTI as well as correct for simple head motion. Second, b_0_ image extraction and brain extraction (fractional intensity threshold = 0.2) were performed. Third, a Bayesian Estimation of Diffusion Parameters Obtained using Sampling Techniques (Bedpostx) was used to set up the distribution of fiber orientation at each voxel. In the T1 images, BET was utilized for the brain extraction (fractional intensity threshold = 0.3).

### Network Construction

The brain network contains nodes and edges. To determine the nodes in the network, we selected 90 gray matter regions of the cerebrum with Anatomical Automatic Labeling (AAL), which included 45 regions of cortical and subcortical structures in each hemisphere. Edge definition was accomplished using the connectivity probability between each pair of nodes in the network. Network construction was performed by PANDA (30). The details of network construction were as follows.

#### Node Definition

The procedure was completed following Gong's description (31). Briefly, T1 images were nonlinearly coregistered to the Montreal Neurological Institute (MNI) 152_T1_Template. The inverse warp was obtained from a previous step and the transformation matrixes from T1 to b_0_ were combined to warp the AAL regional mask from the MNI space to the individual T1 space and b_0_ space successively. In total, 90 AAL regions were executed using the procedures described above to establish the seed mask. For each of the seed masks, the remaining 89 regional masks were merged to form the terminal mask.

#### Edge Definition

As aforementioned, probabilistic tractography (FSL 5.09) was used to define the edge of the brain network. For each voxel in the seed mask, 5,000 streamlines were sampled. Each fiber was drawn depending on the distribution of the orientation set up by Bedpostx. The tract was established every 0.5 mm to the other 89 masks and terminated in a terminal mask to prevent tracking in the loop with parameters of 0.2 curvature threshold, 2,000 steps with length of 0.5 mm. Then, the connectivity probabilities from the seed mask to the remaining 89 target masks were established. The probability was defined as weight. After 90 seed masks were performed, the same procedure that was described above was performed, and a 90 × 90 connective matrix was constructed for each participant.

As it is impossible to determine the directionality between node A and node B by probabilistic tractography, we defined the unidirectional connective matrix using Schmidt's description (32), the probabilities of node A and node B were calculated by the average of weight_AB_ and weight_BA_.

### Network Analysis

To describe the topological features of the WM network at a global level, we calculated several global network properties: clustering coefficient (Cp), shortest path length (Lp), efficiency (E), connective strength (S), fault tolerant efficiency (E_loc_), density of network (Density), small world properties, modularity (Q), hierarchy (β), and assortativity (r). All of the global network properties were calculated with the GRETNA toolkit (33) or our custom-made MATLAB program. To rule out spurious connections, a connection was excluded from the connective matrix before calculating the global network properties if it existed in fewer than 20% of the group subjects (24).

#### Global Clustering Coefficient (Global Cp)

The global clustering coefficient is defined as the average of the likelihood of a neighbor-to-neighbor connection. A greater value represented a larger extent of the local interconnectivity of a network. For a network G, the equation is:

$$\text{Global}\;C_{p}=(1/N)\times \{\sum_{A\in G}\left[2/k_{A}(k_{A}-1\right)\sum_{B,k}{\left(\omega_{\text{AB}}\omega_{\text{Bk}}\omega_{\text{kA}}\right)}^{\frac{1}{3}}]\}$$

k_A_ is the degree of node A and ω_AB_ is the weight between node A and node B. N = 90.

#### Global Shortest Path Length (Global Lp)

The global shortest path length is defined as the average of all of the shortest lengths between each pair of nodes in the network. A smaller value represented a faster transfer speed of information in the brain. For a network G, the equation is:

$$\text{Global}\;L_{p}=[1/N(N-1)]\times \sum_{A\neq B\in G}L_{\text{AB}}$$

L_AB_ is the shortest path length between node A and node B and N = 90.

#### Small World Properties

Before the calculation of the small-world property, 1,000 random networks that maintained the same nodes and edges as the original network but also maintained the differences in distribution were generated. The small-world properties consisted of the normalized global clustering coefficient Gamma (γ) and the normalized global shortest path length Lambda (λ), which represented the means of 1,000 random network global clustering coefficients and the global shortest path length respectively.

$$\begin{array}{ccc} \gamma =\text{Global}\;C_{p}^{\text{real}}/\text{Global}\;C_{p}^{\text{rand}} & \;\ldots & \lambda =\;\text{Global}\;L_{p}^{\text{real}}/ \end{array}\text{Global}\;L_{p}^{\text{rand}}$$

The small-world measurement sigma (σ), where σ = γ/λ, γ > 1, λ ≈ 1 and σ > 1, indicate the existence of small-world properties.

#### Network Connective Strength (Global S)

The connective strength of node A is defined as the sum of the connective weight, which is the weight that directly connects to node A. Network connective strength is the average of all of the nodal connective strengths in the network. For a network G, the equation is:

$$S=(1/N)\times [\Sigma_{A\in G}(\Sigma_{B\in G}\omega_{AB})]$$

The variable ω_AB_ represents the weight between node A and node B. A greater network connective strength is represented by a greater connection connective strength in the network.

#### Global Efficiency (E_glob_)

The global efficiency is represented as the information transfer efficiency of the network. For a network G, the equation is:

$$E_{glob}=[1/N(N-1)]\times \sum_{A\neq B\in G}1/L_{AB}$$

where L_AB_ is the shortest path length between node A and node B.

#### Global Fault Tolerant Efficiency (E_loc_)

The E_loc_ of node A is defined as how much of the network is fault-tolerant when the first neighbors of node A are removed from the network. The global E_loc_ is the average of the nodal E_loc_ values in the network. A greater global E_loc_ represents a greater fault tolerance of the network. For a network G, the equation is:

$$E_{loc}=(1/N)\times \sum_{A\in G}E_{glob}(G_{A})$$

#### Density of Network (Density)

The density of a network is the fraction of present connections to possible connections. In this study, the possible connections are equal to N(N-1)/2.

#### Hierarchy (β)

Many real networks, particularly brain networks, commonly share a natural topological property that is called a hierarchical organization. In a hierarchical network, the low-degree nodes in the graph typically exhibit a higher clustering coefficient compared with high-degree nodes and vice versa, yielding an efficient network communication. For reasons of the strict scaling law and scale-free properties, the hierarchical coefficient could be described by the distribution of the ratio, the equation is:

$$C∼k^{-\beta }$$

in which the C represents the clustering and k indicates the degree of a node in a network β; the coefficient of hierarchical organization was calculated by fitting a linear regression with the ratio of the log-transformed C to the log-transformed k.

#### Assortativity (r)

Assortativity is defined as the degree to which one node tends to connect with other similar nodes in the network. In this study, we calculated assortativity with the Pearson correlation coefficients of the connective strength between each pair of linked nodes in the network, as described by Leung and Chau (34). The equation is:

$$r^{\omega }=\frac{H^{-1}\Sigma_{\phi }(\omega_{\phi }\Pi_{A\in F(\phi )}k_{A}-{\left[\frac{H^{-1}}{2}\Sigma_{\phi }(\omega_{\phi }\Sigma_{A\in F(\phi )}k_{A})\right]}^{2}}{\frac{H^{-1}}{2}\Sigma_{\phi }(\omega_{\phi }\Sigma_{A\in F(\phi )}k_{A}^{2}-{\left[\frac{H^{-1}}{2}\Sigma_{\phi }(\omega_{\phi }\Sigma_{A\in F(\phi )}k_{A})\right]}^{2}}$$

where the edge of the network is sorted by ascending values, H is the total weight of all the edges in the network, ω_φ is the weight of the φth edge and F(φ) is the pair of nodes that is connected by the φth edge. If M represents the total degree of the network, then φ = 1~M.

#### Modularity (Q)

To describe the patterns of integration within the module and the segregation between them, modularity was calculated. Modularity is also called Q, a larger Q indicates more connection than was expected within the chosen communities. The definition of the modularity pattern was determined by a method that was previously described by Reichardt and Bornholdt (35) that aims to maximize the number of within-group edges and minimize the number of between-group edges. The equation is:

$$\text{Q}=\frac{1}{2\text{m}}\sum_{\text{AB}}\left[{\text{E}}_{\text{AB}}-\frac{k_{A}k_{B}}{2\text{m}}\right]{\text{δ}}_{c_{A},c_{B}}$$

where m refers to the total weight of the edges in the network, E_AB_ is the connectivity between node A and node B; k_A_ is the degree of node A; δ_AB_ is the Kronecker delta symbol; C_A_ is the mode to which node A is assigned.

### Statistical Analyses

The statistical analysis was performed with Statistical Package for Social Sciences software version 22.0 (SPSS IBM, Chicago, Illinois, USA). The demographic and clinical variables were analyzed using a Chi-square (*χ^2^*) test for the categorical variables and two-sample *t*-tests were used for the continuous variables. Further, we used a general linear model (GLM) with diagnosis as the independent factor and age, gender, and education years as covariates to determine the group differences in neuropsychological scores and WM global network properties. To determine the group differences in the serum levels of TRP, KYN, and KYNA, we used a GLM with diagnosis as the independent factor and age, gender and BMI (36) as covariates. The level of significance was set as a two-tailed *P* value of 0.05.

To determine the significant differences in the subnetwork connection between the LLD and the HC groups, network-based statistics (NBS) were utilized (37, 38). First, the *t* test statistical threshold was chosen by the primary threshold (*P* < 0.01). Second, a two-sample one-tail *t* test (LLD < HC and LLD > HC) was computed for difference in the edges between the LLD and HC groups. A set of suprathreshold links was constructed according to the statistical threshold. Third, a connected graph component was determined by breadth search and the component size M was established by the sum of the test statistical values across all connections in the component. A permutation test (5,000 permutations) was used to correct for multiple comparisons (*P* < 0.05 with a FWE rate approach). The size of the largest component was recorded in each permutation and generated a random component size distribution. Finally, the correct *P* value was determined from the position of M in the random component size distribution. A significantly different subnetwork between the LLD and HC groups was obtained. The connective strength of the NBS subnetwork was calculated.

Furthermore, Pearson correlation was applied to determine relationships between DG performance and clinical variates and KP metabolism, DG performance and WM network properties, and KP metabolites and WM network properties in LLD and HC groups respectively.

## Results

### Demographic and Clinical Characteristics

There is no significant difference in the gender, age, BMI, education years, or MMSE scores between the LLD and HC groups (all *P* > 0.05). The HAMD-17 scores and DG performance were significantly different between these two groups (both *P* < 0.05). Serum concentrations of TRP and the KYN/TRP ratio were significantly different between these two groups (both *P* < 0.05). The details are shown in **Table 1**. In the LLD group, 3 patients were medication-free, 2 patients received serotonin noradrenaline reuptake inhibitors, 15 patients received selective serotonin reuptake inhibitors, no patients received tricyclic antidepressants, 8 patients received noradrenergic and specific serotonergic antidepressants, and 20 patients received benzodiazepines as a combination treatment within the last 3 months.

**Table 1:** Demographic and clinical characteristics, TRP, KYN, and KYNA) levels of MDD patients and HCs.

	LLD	HCs	Statistics	*P* value
	(*n* = 34)	(*n* = 36)	(*z/t*)	
Gender (male/female)	11/23	6/30	2.340^a^	0.126
Age (years)	65.000 ± 5.592	65.830 ± 7.296	0.534^b^	0.595
Education (years)	8.764 ± 3.447	9.681 ± 3.366	1.125^b^	0.265
BMI	21.867 ± 2.918	22.519 ± 2.779	0.957^b^	0.342
MMSE	25.680 ± 2.184	26.670 ± 2.098	2.900^c^	0.093
HAMD-17	9.560 ± 7.439	1.390 ± 2.979	34.085^c^	<0.001
DG	682.350 ± 266.823	502.780 ± 171.524	11.236^c^	0.001
TRP (ng/ml)	11,194.851 ± 2,141.914	12,598.841 ± 1,793.315	8.053^d^	0.006
KYN (ng/ml)	326.566 ± 67.562	329.790 ± 44.917	0.004^d^	0.985
KYNA (ng/ml)	7.068 ± 2.328	7.691 ± 2.649	0.999^d^	0.321
KYN/TRP	0.030 ± 0.009	0.026 ± 0.004	4.575^d^	0.036
KYNA/KYN	0.021 ± 0.006	0.023 ± 0.007	1.236^d^	0.270

LLD, late-life depression; HCs, healthy controls; BMI, body mass index; MMSE, Mini-Mental State Examination; HAMD-17, 17-item Hamilton Depression Scale; DG, Dictator Game; TRP, tryptophan; KYN, kynurenine; KYNA, kynurenic acid; KYN/TRP, kynurenine to tryptophan ratio; KYNA/KYN, kynurenic acid to kynurenine ratio.

^a^P-value was obtained using a Chi-square (χ^2^) test.

^b^P-values were obtained using two-sample t-tests.

^c^P-values were obtained using GLMs with diagnosis as the independent factor and gender, age and education as covariates.

^d^P-values were obtained using GLMs with diagnosis as the independent factor and gender, age and BMI as covariates.

### Global Network Properties

The global topologies of the WM network are shown in **Table 2**. Small-world organization (γ > 1, λ ≈ 1, σ > 1) was observed in both the LLD and HC groups' connectivity networks. Intergroup differences were found in the global Cp, global Lp, small-world properties, global S, E_glob_, and density between the LLD and HC groups (all *P* < 0.05).

**Table 2:** Global network properties of white matter network of LLD patients and HCs.

	LLD	HCs	Statistics	*P* value
	(*n* = 34)	(*n* = 36)	(*t*)	
Global Cp	0.004 ± 0.0001	0.005 ± 0.0001	13.707	<0.001
Global Lp	45.513 ± 3.525	47.148 ± 3.348	6.553	0.013
Gamma, γ	2.072 ± 0.235	2.236 ± 0.229	11.355	0.001
Lamda, λ	1.266 ± 0.014	1.276 ± 0.014	11.950	0.001
Sigma, σ	1.635 ± 0.172	1.751 ± 0.170	10.321	0.002
Global S	0.416 ± 0.033	0.402 ± 0.027	6.190	0.015
E_glob_	0.022 ± 0.001	0.021 ± 0.001	6.619	0.012
E_loc_	0.022 ± 0.001	0.022 ± 0.001	2.944	0.091
Density	0.562 ± 0.021	0.536 ± 0.018	37.823	<0.001
β	−0.345 ± 2.103	−0.0413 ± 2.032	0.682	0.412
r	−0.029 ± 0.009	−0.031 ± 0.012	0.345	0.559
Q	0.593 ± 0.011	0.593 ± 0.117	0.255	0.615

LLD, late-life depression; HCs, healthy controls; Cp, clustering coefficient; Lp, shortest path length; γ, normalized clustering coefficient; λ, normalized shortest path length; σ, small-worldness; S, network connective strength; Eglob, global efficiency; Eloc, fault tolerant efficiency; Density, density of network; β, hierarchy; r, assortativity; Q, modularity.

P-values were obtained using GLMs with diagnosis as the independent factor and gender, age and education years as covariates.

### NBS Analysis

**Figure 1:**
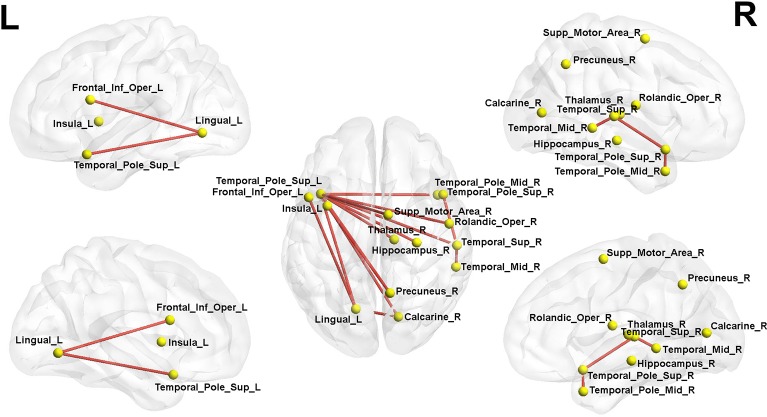
Network-based statistics (NBS) analysis showed a single subnetwork composing of 14 nodes and 15 edges in LLD patients compared with the HCs. Red lines represent the edges of the subnetwork connections. L, left; R, right; LLD, late-life depression; HCs, healthy controls. The nodes and connections were mapped using Brain Net Viewer software (http://www.nitrc.org/projects/bnv/).

We found that the subnetwork strength detected by NBS of the LLD group was 0.307 ± 0.0248, the NBS subnetwork strength of HC group was 0.282 ± 0.0214. The difference of the NBS strength measurements between these two groups was significant (*F* = 24.625, *df* = 1, *P* < 0.001). We found a disrupted subnetwork composing of 14 nodes and 15 connections in LLD group (HC < LLD, *P* = 0.036). The involved nodes in this NBS-based subnetwork mainly included the frontal (Frontal_Inf_Oper_L), parietal (Rolandic_Oper_R, Supp_Motor_Area_R), paralimbic (Insula_L, Hippocampus_R, Calcarine_R, Lingual_L, Precuneus_R, Thalamus_R), and temporal (Temporal_Sup_R, Temporal_Pole_Sup_L, Temporal_Pole_Sup_R, Temporal_Mid_R, Temporal_Pole_Mid_R) gyrus. This NBS's subnetwork is shown in **Figure 1**.

### Correlation Analysis

A partial correlation was conducted with the DG task, KYN/TRP ratio and KYNA/KYN ratio, gender, age, education, and BMI as covariates. We considered KYN/TRP ratio and the KYNA/KYN ratio could represent the changes of KP metabolism most. We found that, in the LLD group, the DG task was correlated with KYNA/KYN ratio (*r* = −0.371, *P* = 0.037). In addition, the HC participants who performed the DG task also showed a trend toward the KYNA/KYN ratio (*r* = −0.334, *P* = 0.053). Furthermore, a partial correlation was conducted with the MMSE, HAMD-17, KYN/TRP ratio, and KYNA/KYN ratio, gender, age, education and BMI as covariates. In LLD group, HAMD-17 was significantly correlated with KYNA/KYN ratio (*r* = 0.507, *P* = 0.003). In HC group, HAMD-17 was significantly correlated with KYN/TRP ratio (*r* = 0.362, *P* = 0.049). No significant relationships were observed among DG task, MMSE and HAMD-17 scores in LLD group and HC groups respectively.

A partial correlation was performed between the WM network properties and DG task, gender, age, and education years as covariates. In LLD group and HC group, no significant correlations were observed between WM network properties and DG task. Moreover, a partial correlation was performed between the WM network properties and KP metabolism, gender, age, education and BMI as covariates. KYNA/KYN ratio was significantly correlated with Density (*r* = −0.576, *P* < 0.001, *q* = 0.024), small world properties [γ (*r* = 0.467, *P* = 0.007, *q* = 0.028) and σ (*r* = 0.442, *P* = 0.011, *q* = 0.033)] and Q (*r* = 0.468, *P* = 0.007, *q* = 0.028) in HC group. No significant correlations were observed between KP metabolism and network properties in LLD group.

A partial correlation was performed between the DG task and the WM subnetwork strength detected by NBS, gender, age, and education as covariates. We did not observe any significant correlations between the DG task and the WM subnetwork strength in the LLD group and HC group respectively. In addition, a partial correlation was conducted with the KYN/TRP ratio, KYNA/KYN ratio, and the NBS subnetwork strength, gender, age, education, and BMI as covariates. We did not observe any significant correlations between the KYN/TRP ratio, KYNA/KYN ratio and the WM subnetwork strength in the LLD group and HC group, respectively.

## Discussion

To the best of our knowledge, the current study is the first to explore the neurobiological mechanism and structural brain network that underlies altruism in older people with depression. We compared the altruistic behavior in the DG task, serum concentrations of TRP, KYN, and KYNA, and the WM network topological features between the LLD and HC participants. The preliminary findings of the current study were as follows: (1) LLD participants exhibited a higher altruism level than HC participants; (2) in LLD group, DG performance was negatively correlated with the KYNA/KYN ratio, while HAMD-17 was positively correlated with KYNA/KYN ratio; (3) in their global network properties, the LLD participants displayed decreased global Cp, global Lp, and small-world properties, increased global S and E_glob_ and density compared to the HCs; (4) KYNA/KYN ratio was correlated with density, small world properties (γ and σ) and Q in HC group.

In the current study, LLD participants demonstrated a higher level of altruism. If people are motivated by self-interest in response to the passive recipient in the DG, the proposer will offer the smallest amount, even zero, as this is the optimal, ‘smartest' decision they can make (8). Previous studies showed that MDD patients would not exhibit higher altruism due to the stress from the cost of helping others (9), reducing the use of task representations and hindering performance during exploratory decision-making (39) and undervaluation of actions leading to rewarding outcomes (40). However, the LLD patients shared more than their fair share in this study. The ability to make a ‘smart’ decision becomes vulnerable in patients with neuropsychiatric disorders and in the aftermath of chronic stress; this phenomenon has been observed in the prefronto-striatal circuit dynamics in rodents exposed to chronic stress (41). However, the level of dopamine in the ventral striatum is one of vital factors that influences altruism decision (8). Furthermore, we found that the breakdown of KYN was closely related to depression, had an effect on altruistic decision-making in the LLD patients of the current study.

The KYNA/KYN ratio had an impact on DG performance in the LLD participants. The KYNA/KYN ratio is closely related to cognition. In the presence of inflammation, the formation of QUIN is enhanced, which result in excitotoxic neurodegenerative changes and cognitive deficits. In addition, KYN/TRP ratio was increased inpatients with LLD, enhanced KYN synthesis might induce an abnormality in KYNA formation that is also harmful to cognition. If KYNA formation is beyond the physiological level, it can lead to glutamatergic hypofunctioning. KYNA is an antagonist of all ionotropic excitatory amino acid receptors; specifically, KYNA has a higher affinity for the alpha-7-nicotinic acetylcholine receptor than for the NMDA-R, this disrupts auditory sensory gating. Furthermore, the level of KYNA produces is associated with the extracellular dopamine concentrations, this influences dopaminergic neurotransmission (17, 42). However, the dopamine in the ventral striatum is involved in the reward process, by which people feel rewarded from helping others (7, 8, 43). Briefly, in the current study, we found that an imbalance of KYN metabolism would have a negative impact on altruistic behavior, which might be caused by cognitive deficits or abnormal dopamine level. Future study should aim at the role of cognitive function and the effects of the level of dopamine in the altruistic behavior of LLD.

In addition, we found that the serum level of TRP was lower in the LLD patients than in the HCs, reflecting the conversion of TRP to KYN and its insufficient quantity for 5-HT synthesis. These results are in line with the mechanism of depression. According to our partial correlation analysis, we found that HAMD-17 was positive associated with KYNA/KYN ratio in LLD. It is not consistent with our previous findings in late-onset depression, which showed negative correlated with depressive factor (15). In the current study, we did not divide HAMD-17 into serval factors, due to the limited sample size. This might be the reason of the opposite finding in the current study. However, the main topic of this study was to explore whether KP metabolism and WM deficits have an effect on altruistic behaviors in LLD. The association of KP metabolism and LLD had been fully discussed in our previous studies (15, 44), which was not our main concern in this study. Thus, we are confident with our current findings in altruistic behavior in LLD. However, a larger sample study is still needed in future study.

In the analysis of their global network properties, LLD participants displayed decreased Cp, Lp and small-world properties, increased strength, E_glob_ and density compared with the HC participants. We determined that LLD participants presented with an increased global network efficiency rather than a decreased regional efficiency, as suggested by the findings of the previous study (45). In addition, some network properties were associated with KYNA/KYN ratio in HCs. While, such relations were lacked in LLD and no difference was found between LLD and HC in KYNA/KYN ratio, indicated that the WM damage of LLD might be not relevant with the changes of KP. Notably, left superior temporal pole was the important node based on NBS analysis. According to the previous studies, superior temporal pole involved in process of depression (46), cognitive impairment (47), and decision-making (48). To some extent, LLD patients have a higher risk of cognitive deficits. The current study hinted that superior temporal pole played an important role in the abnormal altruistic behavior in LLD, but the specific mechanism is needed to be explored in further experiments.

Several limitations must be addressed here. First, we did not quantifiably detect the level of dopamine and calculate relationships among the altruistic, levels of dopamine and KP metabolites in this study. We will make our efforts in future studies. Then, the sample size was small, but it was still statistically significant. We will increase the sample size in future research.

## Conclusion

Our exploratory findings showed that LLD participants exhibited a higher level of altruism than the HC participants. The LLD participants manifested profound shifts in KP that influenced their altruistic behavior. KYNA/KYN ratio influenced altruistic behavior and depressive severity in LLD patients, WM global network properties in HCs.

## Data Availability Statement

The data used to support the findings of this study are available from the corresponding author upon request.

## Ethics Statement

The studies involving human participants were reviewed and approved by the ethics committee of the Affiliated Brain Hospital of Guangzhou Medical University (Guangzhou Huiai Hospital). The patients/participants provided their written informed consent to participate in this study.

## Author Contributions

YW and NM were responsible for the data collection, data analysis. YW was responsible for writing the drafts of manuscript. YN supervised experiment design, and the drafts of the manuscript. XW and JL were involved in revising the drafts. All authors approved the final version of the manuscript.

## Funding

This work was supported by Science and Technology Plan Project of Guangdong Province (No.2019B030316001); Guangzhou Municipal Psychiatric Disease Clinical Transformation Laboratory (No. 201805010009); Key Laboratory for Innovation platform Plan, Science and Technology Program of Guangzhou, China; Guangzhou municipal key discipline in medicine (2017-2019). The funding agency had no role in study design, data collection, analysis, decision to publish, or preparation of the manuscript.

## Conflict of Interest

The authors declare that the research was conducted in the absence of any commercial or financial relationships that could be construed as a potential conflict of interest.
